# Therapeutic Values of General Anesthetics: From Developmental Neurotoxicity to Neurotherapeutic Agents

**DOI:** 10.2174/011570159X388095251029043707

**Published:** 2025-11-29

**Authors:** Tianyun Zhao, Xinying Guo, Ziwen Shi, Haiping Xu, Shiqi Deng, HangChao Tang, Hui Cai, Xingrong Song

**Affiliations:** 1 Department of Anesthesiology, Guangzhou Women and Children’s Medical Center, Guangzhou Medical University, Guangzhou, China

**Keywords:** General anesthetics, neuroplasticity, neurotoxicity, neuroprotective, neurotherapeutic, excitation/inhibition

## Abstract

The development of the central nervous system is characterized by precisely orchestrated, dynamic processes that commence at the embryonic stage and continue throughout postnatal life. Maintaining the balance between excitation/inhibition (E/I) in cortical neuronal circuits is crucial for normal brain function. General anesthetics (GAs) powerfully modulate neuronal activity by enhancing inhibition and/or inhibiting excitability, resulting in temporary loss of consciousness. Therefore, these agents can also induce aberrant neuroplasticity contributing to neurological dysfunction and abnormal behavioural phenotypes, particularly in the developing brain. While this impaired plasticity poses a risk, it also creates an opportunity to treat diseases characterised by abnormal neuroplasticity as core pathologies, such as neuropsychiatric disorders (NPDs). Over recent decades, intense investigations have revealed the neuroprotective and psychotherapeutic potential of GAs in treating neurological injuries and NPDs. Although promising, significant challenges remain, including optimizing dosages, administration duration, and intervals for non-anesthetic uses while minimizing adverse effects. Additionally, the molecular mechanisms underlying the dual roles of GAs - as neurotoxic agents and neurotherapeutic tools - require further elucidation. This review explores developmental neuroplasticity during critical periods, the mechanisms of GAs' action on neural circuits, and the current understanding of their neurotoxic and neuroprotective effects based on alterations in neuroplasticity. Furthermore, we highlight the therapeutic potential of GAs for neurological disorders with impaired neuroplasticity as the core pathological mechanism and propose directions for future research to unlock their full clinical utility.

## 1 INTRODUCTION

General Anesthetics (GAs) are widely used in surgical and diagnostic procedures to induce amnesia, unconsciousness, analgesia, and immobility. Since the first public demonstration of ether anesthesia in 1846, various chemically distinct GAs have been developed, each with unique pharmacological targets and mechanisms of action [1, 2]. These agents primarily modulate neural activity by interacting with GABAergic and glutamatergic receptors, altering the excitability of neural circuits, leading to GAs being regarded not just as drugs that temporarily interrupt consciousness, but also as modulators of neuroplasticity [3-5]. Although GAs are indispensable in modern medicine, their effects extend beyond temporary unconsciousness and can induce long-lasting structural and functional changes, particularly in the developing and aging brain [6, 7]. The vulnerability of the developing brain to GAs has raised concerns about neurotoxicity, as evidenced by clinical and preclinical studies [6-11]. The United States Food and Drug Administration issued a precautionary communication on GA use in patients aged 3 years and under, citing potential risks of long-term neural alterations [12]. However, the same plasticity that makes the developing brain vulnerable to external perturbations also provides opportunities for neuromodulators (*e.g*., GAs) as therapeutic interventions for the diseases that typically occur in the early developmental period before puberty, such as neuropsychiatric disorders (NPDs), neurodevelopmental disorders (NDDs), and neurological injuries. Indeed, emerging evidence suggests that GAs may have neuroprotective and psychotherapeutic potential, particularly in conditions characterized by abnormal neuroplasticity and excitation-inhibition (E/I) imbalances, such as autism and schizophrenia [13, 14]. While previous reviews have addressed the neurotoxicity, neuroprotection, and non-anesthetic clinical applications of GAs, this article aims to provide a unified framework for understanding their therapeutic potential. By focusing on neural circuit E/I balance, we explore how GAs can modulate neuroplasticity to improve neural function under pathological conditions. This review synthesizes recent findings on the dual effects of GAs, emphasizing their developmental and dose-dependent influences, and highlights future directions for research into their therapeutic applications.

## 2 DEVELOPMENTAL NEUROPLASTICITY AND DEVELOPMENTAL VULNERABILITIES

### 2.1 Critical Periods in Development

Here, we will review key events in brain development to identify vulnerable stages following injury or environmental stimuli. The development of the Central nervous system is characterized by a series of precisely orchestrated, dynamic processes, which commence at the embryonic stage and continue throughout postnatal life. These processes include neurogenesis, differentiation, neuronal migration, and synaptogenesis, all of which are fundamental for the establishment and refinement of neural networks. For instance, neural tube formation starts approximately on gestational day (GD) 9-9.5, with neurogenesis reaching its peak at approximately gestational day 14 in mice. Atrogliogenesis, a process that occurs after neurogenesis, does not peak until shortly after birth, around postnatal days (PND) 2-3 [15, 16]. Microglia migrate into the brain during a specific temporal window that spans both neurogenesis and astrogliogenesis, with the onset of migration occurring at approximately GD 9 in mice [15, 17, 18]. Programmed cell death (PCD) of neural cells, believed to be essential for the establishment and maintenance of the nervous system, is found during most stages of development and ultimately results in the elimination of 50% or more of the initial neuronal pool [19, 20]. The most prominent PCD is observed during the process of synaptogenesis in postmitotic neurons. In rats, the peak of postnatal synaptogenesis occurs at PND 7, and is essentially complete by the age of two weeks [19, 20].

These highly programmed series of processes are linked to the concept of 'critical' or 'sensitive' periods hallmarks of early development. These periods are highly sensitive to both intrinsic cues and external stimuli, which can lead to either a poor outcome or a window of opportunity for treatment [21]. Indeed, disruption of critical periods of developing has been implicated in various neurodevelopmental disorders and mental illnesses, most of which onset before adolescence [22].

### 2.2 Developmental Plasticity

Neuroplasticity is an intrinsic property of the (CNS), reflecting its capacity to respond dynamically to the environment and experience by modifying neural circuitry [23]. Accordingly, “neuronal plasticity” includes morphological and functional changes in neuronal networks, including changes in neuronal connectivity (formation of connections), the generation of new neural cells and brain tissue (neurogenesis, gliogenesis, angiogenesis, *etc*.), and neurobiochemical changes (neurotransmitters, pre- and postsynaptic activities) [24]. Developmental neuroplasticity, a complex, genetically encoded, sequenced, maturational, and time-dependent process, is an umbrella term encompassing fundamental changes in neurogenesis, migration, the formation of connections, and the specialization of neural circuits, and is strictly regulated by intrinsic homeostatic mechanisms and influenced by environmental cues [21, 25, 26]. Among these, synaptic plasticity is the most important form of neuroplasticity during development, allowing the formation of precise neural connectivity *via* the process of synaptogenesis and pruning [27]. Multiple mechanisms that influence the dynamics of synaptic connections and neural circuitry interact to maintain a delicate balance between developmental neuroplasticity and achieve a reasonable functional state that is “neither too rigid nor too flexible” [28]. Factors responsible for modification of synaptic plasticity including spontaneous activity, environmental stimulation, synaptic structure (dendrites, synapses and axons), synaptic proteins (pre- and postsynaptic proteins), neurotransmission (GABAergic and Glutamatergic transmission, E/I balance, *etc*.) and epigenetic factors [27, 29]. Moreover, macroglia (including astrocytes and oligodendrocytes) and microglia, two major forms of glial cells, are commonly considered tissue-supporting cells and “immune cells of the CNS”, respectively [30]. Recent findings have revealed their active involvement in a variety of key developmental processes and neuroplasticity, including neuro-/gliogenesis, blood-brain barrier (BBB), axonal outgrowth, angiogenesis, synaptogenesis, synaptic plasticity and neurotransmission [30, 31]. For example, microglia-dependent regulation of synaptic plasticity during the development and throughout the lifetime is mainly executed *via* synaptic elimination [32]. Due to their critical instructive roles in these processes, dysfunction of microglia or astrocytes during brain development could contribute to NDDs and potentially even late-onset neuropathology [17, 30].

At the molecular level, various neurotransmitters and their receptors are expressed in spatio-temporal patterns to influence early developmental events and play an important role in wiring neural circuits and brain plasticity during critical developmental windows. For example, N-methyl-D-aspartate (NMDA) receptors develop before alpha-amino-3-hydroxy-5-methyl-4-isoxazolepropionic acid (AMPA) receptors to provide the basic platform for network formation, and finally AMPA receptors develop at the right time for experience-dependent long-term potentiation (LTP) and long-term depression (LTD) plasticity [33]. Synaptic transmission is mainly mediated by presynaptic exocytosis of synaptic vesicles containing neurotransmitters, which are detected by specific postsynaptic receptors [34]. It has been well documented that both glutamatergic and GABAergic systems play critical roles in regulating developmental processes and neuroplasticity, including neuronal survival, neuronal differentiation, synaptogenesis, and neuroplasticity in the immature brain [35-38]. Given the involvement of these neurotransmitter systems in normal development, it has therefore been speculated that the developing brain may be much more susceptible to the adverse effects of agents that target NMDA and/or GABAA receptors (*e.g*. GAs and alcohol) than the adult brain. Furthermore, Structural and functional plasticity is associated with a series of neurotrophic signaling pathways, including Ca^2+^-dependent calmodulin-dependent protein kinase (CaMK2), the brain-derived neurotrophic factor/tropomyosin receptor kinase B (BDNF/TrkB), and the mitogen-activated protein kinase/extracellular signal-regulated kinase (MAP/ERK) pathway [39, 40]. These signaling pathways, amongst others, are known to play a critical role in neuroplasticity by activating downstream signaling cascades to mediate neuronal survival, neuronal proliferation, and neural architectures. Taken together, these studies have demonstrated that the spatio-temporal evolution of the neurotransmitter system is coupled to major developmental events, and that any alteration in transmission would therefore affect developmental neuroplasticity. In the context of normal developmental conditions, neuroplasticity is generally regarded as an adaptive process and beneficial; however, the outcome is not always beneficial and can lead to maladaptive outcomes depending on the type and extent of the neuropathogenic process, the stage of its occurrence, and the homeostatic regulatory mechanisms. There is no doubt that each milestone of neurodevelopment is important, and its window of vulnerability or fragility provides a starting point for understanding the development and severity of neuropsychiatric and NDDs. Notably, this may also provide insights into their potential therapeutic role in nervous system plasticity and regeneration.

## 3 MECHANISMS OF ANESTHETIC ACTION ON THE DEVELOPING BRAIN

### 3.1 Commonly Used Anesthetics and Their Mechanisms

General anesthetic agents, widely used both in clinical medicine and in neuroscience research, produce the anesthetic state, including anxiolysis, sedation, unconsciousness, amnesia, myorelaxation, and analgesia. In neuroscience research, GAs is used to explain the generation of conscious perception, the neural mechanisms of sleep and arousal, and to investigate the cortical circuits that generate rhythmic group activity [4]. Furthermore, many conflicting studies on the neurotoxic and neuroprotective effects of GAs have emerged, which have confused many healthcare practitioners and researchers [41]. Therefore, understanding the molecular mechanisms of action of GAs is critical to understanding the paradoxical results in neuroscience, especially the neurotoxicity and neuroprotective effects of GAs. However, research into the molecular actions of GAs has been a long and winding road, beginning with the success of ether anesthesia by William T. Morton in 1846 and continuing through to the lipid theory of anesthesia prompted by the work of Meyer and Overton in 1900 [42, 43]. More recently, research into the mechanisms of anesthesia has focused on ion channels in the membranes of nerve cells [3, 44-46]. However, it is not clear which of these molecular targets are most relevant for mediating the effects required for clinical anesthesia and neuroprotection, which cause unwanted side effects, and which major brain regions and receptors are targeted by different concentrations of general anesthetics. We will describe the neuronal systems involved in mediating clinically relevant effects of GAs and review the cellular and molecular mechanisms of GAs for altering synaptic transmission and plasticity.

In the late 19^th^ century, Claude Bernard proposed that several structurally unrelated volatile compounds, including ether, chloroform, and nitrous oxide, acted through a common molecular mechanism [47]. By the 20^th^ century, Meyer and Overton proposed the famous lipid theory, which holds that general anesthetics cause structural changes in the lipid bilayer by dissolving into nerve cell membranes. This theory was based on their discovery of a correlation between lipid solubility and anesthetic potency. On the basis of this general non-specific theory, Meyer concluded that all chemically unrelated lipid-soluble agents have an anesthetic effect [42]. However, Franks and Lieb showed in a series of seminal studies that general anesthetics actually act directly with proteins. Furthermore, non-specific actions cannot explain the differences in anesthetic potency between the optical isomers of several anesthetics [48], and lipid perturbations due to changes in temperature do not produce anesthesia, as would be predicted by the lipid theory of anesthetic action [47]. As these further experimental findings which were inconsistent with a non-specific action within lipid membranes, the lipid theory gradually fell by the wayside.

Over the past few decades, research on the mechanisms of GAs has focused on ion channels in the cell membranes of various cells of the CNS. There have been several excellent reviews describing the role of various types of ion channels in the anesthetic state produced by different GAs. For example, Campagna *et al.* provide a comprehensive review of the mechanisms of action of inhaled anesthetics, which lists more than 30 types of ion channels that have been found to be affected within clinically relevant concentrations [5]. In addition to GABA_A_ receptors, which have received the most attention as anesthetic actions, other ion channels probably involved in the actions of volatile anesthetics at clinically effective concentrations include both the superfamily of “cysteineloop” neurotransmitter receptors (nicotinic acetylcholine, serotonin type 3, GABA_A_, and glycine receptors) and the NMDA receptors or AMPA receptors [5]. Rudolph *et al*. focused on the molecular mechanism and neuroanatomical substrates of modern intravenous anesthetics, including propofol, etomidate, and benzodiazepines (BDZs). They described the neuronal systems involved in mediating different clinically relevant actions of GAs and discussed the hypothesis that different components of anesthetic state arise from the action of a single drug in different parts of the CNS (*e.g*. cerebral cortex, thalamus, and brainstem) [3]. Diao *et al*. summarized evidence for site-specificity of GAs in different brain regions and receptor populations [49]. To sum up, whether inhaled or intravenous GAs, although structurally and mechanistically distinct, GAs inhibit excitatory and/or enhance inhibitory synaptic transmission principally by modulating the function of GABAergic or glutamatergic synapses. Synaptic signaling proteins, including ligand- and voltage-gated ion channels, are targeted by general anesthetics to modulate synaptic mechanisms, including presynaptic neurotransmitter release, postsynaptic receptor signaling, and dendritic spine dynamics, thereby producing their characteristic acute neurophysiological effects. Indeed, researches have demonstrated that GAs are involved in the regulation of synaptic transmission and synaptic plasticity through effects on long-term potentiation (LTP) and long-term depression (LTD), which are thought to represent the cellular substrates of learning and memory in the brain [50-54]. The actions of GAs on receptors are not limited to individual neurons since each neuron is closely cemented to other neurons through specialized structures termed synapses so that information can be quickly relayed throughout the nervous system. It is therefore rational that GAs, affecting the receptor activity of a specific neuron, will, in turn, modulate neural connections and E/I balance in the brain [45]. The balance between excitatory and inhibitory neurotransmission is a cornerstone of neural network function, particularly during neurodevelopment [55-57]. Excitatory signals, predominantly mediated by glutamatergic pathways, and inhibitory signals, primarily mediated by GABAergic pathways, must be tightly regulated to maintain neural homeostasis and proper synaptic plasticity [57]. Disruptions in this balance have been implicated in various NDDs, including ASD and schizophrenia [58-60]. Emerging evidence highlights that GAs profoundly influence E/I balance. For instance, GAs such as isoflurane and ketamine modulate GABA_A_ and NMDA receptor activities, potentially shifting the balance towards inhibition during critical developmental windows [61-64]. While traditionally associated with neurotoxicity, these effects also suggest therapeutic potential under certain conditions, where controlled modulation of E/I balance might promote neuroplasticity and network remodeling [65-67]. Given its critical role, E/I dysregulation serves as both a marker and a potential target for therapeutic intervention in neurodevelopmental and neuropsychiatric disorders. Future studies should aim to elucidate the context-dependent effects of GAs on E/I balance, leveraging this knowledge to optimize their use as neurotherapeutic agents.

### 3.2 Neurotoxic and Protective effects of GAs

From the above, it can be hypothesized that modulation of synaptic transmission and neural circuits by the GAs can result in both protective and therapeutic effects, especially in specific pathological states, and neurotoxic effects, leading to morphological changes and long-term behavioral abnormalities at the extremes of age. Here, we mainly review the neurotoxicity that occurs after exposure to general anesthetics in the early stage, while neurotoxicity in the elderly has been covered in other excellent reviews [68-71].

Today, in addition to anesthesia requirements for surgical procedures, general anesthesia is increasingly used for noninvasive and minimally invasive diagnostic procedures (*e.g*., radiology and endoscopy) and therapeutic interventions (*e.g*., electroconvulsive therapy and radiation therapy) that require deep sedation, particularly in pediatric patients. Concerns over the potential adverse effects of exposures to commonly used anesthetics/sedatives after the finding that exposure to the NMDA receptor antagonists, including the widely used anesthetic ketamine, for a few hours caused widespread apoptosis in the late fetal or early neonatal rat brain [72]. Subsequent studies have confirmed that similar patterns of neuronal apoptosis also occur after exposure to benzodiazepines, barbiturates, and ethanol, which is already a well-established teratogen that causes reproducible neurotoxic effects [73, 74]. These findings were of great interest to the scientists, prompting them to wonder whether GAs, which are commonly used in clinical settings, have similar toxic effects. In response to this concern, the first paper, published in 2003, demonstrated that a 6 hrs. exposure to a cocktail of clinically relevant anesthetics (midazolam, N_2_O, and isoflurane) caused widespread apoptotic neurodegeneration in the PND 7 rat brain and long-term cognitive deficits [75]. This data has subsequently been reproduced and extended across a wide variety of species using various GAs in common clinical use, including inhaled anesthetics (sevoflurane, isoflurane, desflurane, and N_2_O) [75-82] and intravenous anesthetics (propofol, etomidate, and ketamine) [83-87]. Further, it was demonstrated that neurotoxic sensitivity was greatest at PND 7 (peak of postnatal synaptogenesis) and lowest at PND 14 (end of synaptogenesis) in rodents [88]. This point of view places synaptic development, rather than apoptosis as initially discovered, at the center of the story of anesthetic neurotoxicity. To date, a substantial body of literature has documented a range of indices of neurotoxicity induced by GAs, and has investigated the underlying mechanisms from multiple perspectives. The majority of these studies have focused on the effects of GAs on neuronal cells (glutamatergic neurons and interneurons) [89]. Recently, however, there has been a growing interest in anesthesia-induced neurotoxicity in non-neuronal cells, including glial cells (ependymal cells, astrocytes, oligodendrocytes, and microglia) and the brain vasculature/BBB [90, 91]. Currently, the mechanisms underlying anesthetic-induced neurotoxicity have been extensively explored. In addition to inducing apoptosis *via* both the intrinsic and extrinsic apoptotic pathways, GAs have been shown to suppress the neurogenesis and proliferation [86, 92], alter the migration pattern and differentiation [93-95], disrupt mitochondria integrity and function [75, 96-98], impair glial morphological integrity and function, alter dendritic spine/synapse morphology and density [99-103], and impair the LTP and LTD [104-106]. In addition, these neurotoxic effects involve multiple brain regions (*e.g*., cerebral cortex, hippocampus, and thalamus) and are developmental stage- and exposure dose- and duration-dependent, and are also related to the number of exposures and sex [7, 98]. Currently, consensus on the GAs-induced developmental neurotoxicity is generally accepted, and the most consistent and robust findings of preclinical studies are as follows [9, 107]: (i) Exposure at a particular species-specific developmental window (for both rodents and non-human primates this has typically been in PND 1-7). (ii) Prolonged or multiple exposures rather than single or short episodes. (iii) Exposure to combinations of multiple anaesthetic agents rather than to monotherapy.

The functional consequences of the neuroanatomical, morphological, electrophysiological, and molecular signaling alterations induced by exposure to GAs early in development are primarily cognitive deficits, emotional abnormalities, and motor deficits in a variety of animal models using different behavioral tests [9, 108-112]. The upward trajectory of the clinical trial literature commenced in 2009 with a series of retrospective clinical studies. However, the findings of these studies were relatively inconclusive, with some but not all indicating cognitive changes following early childhood exposure to general anesthesia [113-123]. In response, on December 14, 2016, the U.S. Food and Drug Administration (FDA) issued a “Drug Safety Communication” warning that “repeated or lengthy use of general anesthetic and sedation drugs during surgeries or procedures in children younger than 3 yr old or in pregnant women during their third trimester may affect the development of children’s brains” [12]. At the time, the warning was based only on preclinical studies and retrospective clinical studies with little prospective clinical data. The warning also called for “…additional high-quality research is needed to investigate the effects of repeated and prolonged anesthesia exposures in children, including vulnerable populations”, a wish shared by the medical and scientific communities. The call has sparked a number of rigorously designed clinical trials, many of which have already published results. The General Anesthesia compared to Spinal Anesthesia (GAS) randomized controlled trial evaluated intelligence as the primary outcome and a variety of secondary outcomes [124, 125]. The results of the interim analysis conducted after 2 years and the primary outcome assessment conducted after 5 years showed that only the executive function secondary outcome had 95% CI bounds that favored a worse outcome in the general anesthesia group [124, 125]. In addition, a meta-analysis combining the results of the GAS study with those of other well-designed prospective trials, including the Pediatric Anesthesia NeuroDevelopment Assessment (PANDA) and the Mayo Anesthesia Safety in Kids (MASK) study, revealed that children exposed to general anesthetics exhibited consistent but minor behavioral score differences, with no discernible impact on intelligence [123, 126, 127]. It is noteworthy that this phenotype, characterized by deficits in behavioral function with no difference in cognitive function measures following anesthetic exposure, is similar to that reported in non-human primate studies [109, 112, 128, 129]. Although the preclinical data are compelling, it is important to be aware of the considerable uncertainty in translating these findings in animal models to clinically relevant human scenarios. Moreover, the interpretation of the human data has been more complex, with mixed results from studies in children. These results may help identify a phenotype of injury after anesthesia exposure, but new research approaches are still needed to determine whether a recognized phenotype is caused by the anesthetic drugs or by other factors related to surgery or the perioperative experience [7]. At present, several avenues for clinical research have been proposed. However, whether any of the proposed studies can answer the basic question of “Do anesthetics cause neurodevelopmental deficits in children?” is still under active debate.

## 4 NEUROPROTECTION AND THERAPEUTIC POTENTIALS

It is clear that the developing brain is highly plastic, and that this plasticity is a window of vulnerability as well as a window of opportunity for therapy. Indeed, anesthetic-induced neuroprotection following CNS injury (*e.g*., hypoxia-ischemia) has been intensively investigated over the past four decades. However, the potential of this neuroprotection has been seriously questioned over the past two decades, mainly for reasons that GAs may have unwanted toxic effects on the brain in young infants or the elderly. On the other hand, given their potent modulatory effects on neuroplasticity and E/I homeostasis, emerging evidence suggests that GAs may have neuroprotective and psychotherapeutic potential. In addition to their therapeutic effect on neurological injuries characterized by excess glutamate release-induced excitotoxicity such as Hypoxic-ischemic encephalopathy (HIE) and traumatic brain injury (TBI) which have been well reviewed [41, 130-133], it is worth noting that recent data suggest that GAs may even have neuroprotective and psychotherapeutic potential, particularly in conditions characterized by E/I imbalances and impaired neuroplasticity due to various etiologies including neuropsychiatric disorders and autoimmune neurological disorders such as amyotrophic lateral sclerosis, autoimmune encephalomyelitis, multiple sclerosis, and stiff-person syndrome. Generally speaking, cognitive, emotional, and behavioral responses are based on prior experiences that can begin in the earliest stages of life. Across species, a high degree of neural plasticity in the developing brain is ecologically advantageous for refining neurocircuitry specifically tuned to the demands of the surrounding environment. However, the same capacity for neural alteration can make the developing brain particularly vulnerable. Indeed, developmental dysregulation of emotional memory systems is a principal component of many psychiatric disorders as demonstrated that up to three-quarters of all psychiatric disorders emerge before age 24 [22, 134, 135]. Autoimmune neurological disorders, without a clear developmental origin, are characterized by neuronal and synaptic loss, leading to an imbalance in excitatory and inhibitory synaptic transmission and a wide range of clinical neurological symptoms. Thus, in this section, we focus primarily on the potential therapeutic use of GAs for neuropsychiatric disorders and autoimmune-related diseases, rather than neurological injuries, which have been thoroughly reviewed elsewhere as mentioned above.

### 4.1 Neuroprotection and Therapeutic Potentials in Neuropsychiatric Disorders

Mental disorders are severely debilitating conditions characterized by a combination of symptoms, including abnormal thinking, perceptions, emotions, behavior, and relationships with others, resulting in enormous personal suffering, social and economic burdens [136-138]. Neuropsychiatric disorders are a heterogeneous group of mental disorders that manifest psychiatric symptoms and neuropathological changes attributed to disruptions in the neural circuits involving abnormal neurotransmission and impaired synaptic plasticity [139, 140]. Traditional pharmacological treatments for neuropsychiatric disorders have typically targeted serotonin reuptake, dopamine receptors, and other atypical agents such as bupropion or mirtazapine [141, 142]. Although effective for many patients, these treatments suffer from a number of serious drawbacks, including high rates of non-compliance, limited efficacy in treating negative symptoms and cognitive dysfunction, resistance to treatment, and weeks to therapeutic effect after administration. These limitations have prompted the search for novel therapeutic approaches to reverse the course of the disease. GABAergic and glutamatergic receptors, owing to their crucial importance in synaptic transmission and plasticity during neurodevelopment and their extensive genetic links to neuropsychiatric disorders, are among the most promising targets for new drug exploration [143, 144]. Since GAs are potent modulators of GABAergic- and glutamate receptors, there is an interesting hypothesis that these drugs can restore damaged neuroplasticity and thus have therapeutic effects on patients with mental disorders [145, 146]. However, much of the research in this area is based on clinical serendipitous findings, which are then validated through a series of clinical trials before moving into the laboratory to explore the mechanisms involved. Therefore, in this section, we begin our review with an overview of historical and clinical studies of general anesthetics in the treatment of neuropsychiatric disorders (Table **1**), followed by an overview of possible mechanisms in the laboratory.

The history of GAs in the treatment of mental illness dates back to the 19^th^ century, when narcotherapy was widely used for a wide range of neuropsychiatric disorders [147, 148]. The introduction of narcotherapy for victims of shell shock with acute combat neurosis constituted a major advance in the Second World War, although it is not mentioned in current editions of comprehensive textbooks of psychiatry [149]. The costly solution of anesthesiologists required for the safe administration of the desired state of narcosis may be one of the major reasons for the declining interest in narcotherapy in the non-war era. The resurgence of GAs for the treatment of psychiatric disorders stems from a clinical observational study conducted in 1953. This study indicated that 40 schizophrenic patients administered pentothal showed improvements in social performance comparable to electroconvulsive therapy (ECT) or non-convulsive stimulation under pentothal anesthesia [150]. Similar conclusions were reached in patients with depressive psychosis, that no significant difference was found on the Hamilton Depression Rating Scale between the methohexitone-anesthesia alone group and the group of ECT under the same anesthesia regimen [151]. This finding was repeated in the Northwick Park Electroconvulsive Therapy Trial, 70 patients who met the indications for ECT were randomly assigned either to a course of 8 simulated ECT or to a course of 8 real ECT under identical methohexitone-based anesthesia, and both groups achieved significant improvements in terms of “psychiatrists’ ratings” over a 4-week-long treatment period [152]. However, in a double-blind controlled trial of ECT *versus* simulated ECT under identical thiopentone-based anesthesia twice weekly in patients with depressive illness, the opposite result was obtained [153]. Base on the assumption that the ECT-induced brief period of electrocerebral silence could be a crucial biological determinant for the therapeutic efficacy of ECT, Langer *et al*. found that rapid antidepressant effects following high concentration-based isoflurane narcotherapy (until the presence of electrocerebral silence or burst suppression in Electroencephalography) in 9 out of 11 major depressed patients [154]. And this therapeutic effect has been confirmed by another study with treatment-refractory depressed patients [155]. Following these observations, Langer G *et al*. again conducted a prospective double-blind study comparing this burst-suppression-isoflurane narcotherapy (ISONR group) with ECT under standard isoflurane anesthesia (ECT group) twice a week for a total of 6 sessions in drug-refractory severely depressed women (10 per group). Interestingly, the antidepressant effects during the treatment period were comparable between the two groups, but the rapid antidepressant effects of the first treatment session were only significant in the isoflurane narcotherapy group, whereas patients of the ECT group tended to relapse during follow-up [156]. Moreover, isoflurane antidepressant treatment showed greater improvement in cognitive function than the ECT group [157]. In 2000, the first double-blinded, placebo-controlled trial demonstrated the rapidly antidepressant effects of subanesthetic low-dose ketamine in depressed patients based on the findings of preclinical studies, which implicates the NMDA receptors in the pathophysiology of major depression and NMDA receptor antagonists, including ketamine, have been shown to be effective in animal models of depression [158, 159]. Subsequently, a growing number of trials replicated the robust antidepressant effects of low-dose ketamine in patients with either depressive disorder or bipolar disorder [160, 161]. At the same time, a large number of laboratory studies explored the underlying mechanisms of antidepressants. Among them, the mechanistic target of BDNF-rapamycin 1 (mTORC1) Complex 1 (mTORC1) signaling is critical because it regulates activity-dependent protein synthesis and enhances synaptic plasticity [162, 163]. Moreover, this activation was dose-dependent: it occurred only at sub-anesthetic doses, not at high anesthetic doses, in line with clinical results. It is particularly noteworthy that increasing number of trials report therapeutic effects of GAs in psychiatric disorders in the last decade. For example, several clinical trials demonstrated that nitrous oxide inhalation has rapid and marked antidepressant effects in patients with treatment-resistant depression [164-169]; the intravenous GAs propofol also showed significant antidepressant effects in patients with treatment-resistant depression [170, 171]; and Low-concentration sevoflurane effectively reversed MK801-induced schizophrenia like disease in mice and alleviated schizophrenia patients' symptoms [172]. Moreover, GAs, are often used for mania, delirium, agitation due to various etiologies such as brain injury and psychiatric disorders [145, 173, 174]. Whether GAs can be a practical first-line therapy for the treatment of these scenarios as well as late maintenance therapy, further studies are needed to confirm these findings. It's worth noting that positive allosteric modulator of GABAA receptors brexanolone and ganaxolone, has been approved by FDA for the treatment of postpartum depression in 2019 and of seizures associated with cyclin-dependent kinase-like 5 deficiency disorder in 2022, respectively [175, 176]. AXS-05, oral combination dextromethorphan (NMDA receptor antagonist) and bupropion (σ_1_ receptor agonist), was FDA approved for the treatment of major depressive disorder (MDD) in adults in 2022. And esketamine, a right-handed split of ketamine, intranasal esketamine (Spravato^®^) was approved by the FDA in 2019 to treat adults with treatment-resistant depression [177, 178]. The approval of these drugs bolsters the importance of amino acid neurotransmitter systems (glutamate and GABA) as novel therapeutic targets and represent an exciting breakthrough in contemporary in neuropsychiatric disorders. Currently, several pharmaceutical companies have launched clinical programs to develop the glutamate-targeting agents, such as lanicemine (AZD6765) and traxoprodil (CP-101,606), and GABA-targeting agents, such as SAGE-217, many of which are already in clinical phase II and III as review by Wilkinson *et al*. Sanacora, [144] and McIntyre *et al*. [179].

Neurodevelopmental disorders (NDDs) are defined as a group of conditions with onset in the developmental period that induce deficits that impair functioning. NDDs comprise intellectual disability (ID); Communication Disorders; Autism Spectrum Disorder (ASD); Attention-Deficit/Hyperactivity Disorder (ADHD); Neurodevelopmental Motor Disorders, including Tic Disorders; and Specific Learning Disorders [180]. Genetic research supports the hypothesis that ID, ASD, ADHD, schizophrenia, and bipolar disorder lie on a neurodevelopmental continuum [181-185], and it is now believed that the common end-pathological theory of these diseases is an increase in the ratio between excitation and inhibition, leading to hyper-excitability of cortical circuits [186]. Based on this theory, modulators of E/I imblance, including glutamatergic modulators, such as memantine and GABAergic modulators, such as STX209, bumetanide and allopregnanolone, have been programmed in clinical trials for children and adolescents with ASD [187, 188]. Dexmedetomidine, a selective alpha-2 adrenergic receptor agonist, a sublingual formulation and has been approved in the US for the treatment of agitation associated with schizophrenia and bipolar disorder in adults [189]. Guanfacine, another enteral alpha-2 agonist like dexmedetomidine, was FDA-approved for treating ADHD in 2009, is recommended for ASD co-occurring attention deficit/hyperactivity disorder [190, 191]. Excitingly, a growing body of research suggests that propofol, sevoflurane, Xenon, and ketamine may improve symptoms of autism in animal models [192-194], and clinical studies are underway (*e.g*., NCT03434366, ChiCTR1900027459) and a two-dose, double-blind, placebo-controlled, cross-over pilot trial of intranasal ketamine included individuals with ASD (N = 21) aged 14-29 years, showing no significant impact on the core social impairment assessed by the Aberrant Behavior Checklist Social Withdraw subscale [195]. It is clear that GAs can ameliorate the co-occurring healthy problems of ASD, such as epilepsy, sleep disorders, anxiety, and attention deficit/hyperactivity disorder, but it is still unknown whether GAs can ameliorate the core symptoms of ASD which need further investigation and more clinical trials. In addition, epilepsy, with a high prevalence of psychiatric disorders, is a neurological disorder characterized by a persistent predisposition to recurrent seizures primarily due to the imbalance between excitatory and inhibitory neuronal activity [196]. GAs, such as barbiturates, midazolam, volatile GAs, propofol, and ketamine, are well-established agents for the management of refractory status epilepticus [197-200]. However, whether GAs can be used as routine antiepileptic drugs and their effect on long-term outcome is unclear.

**Fig. (1):**
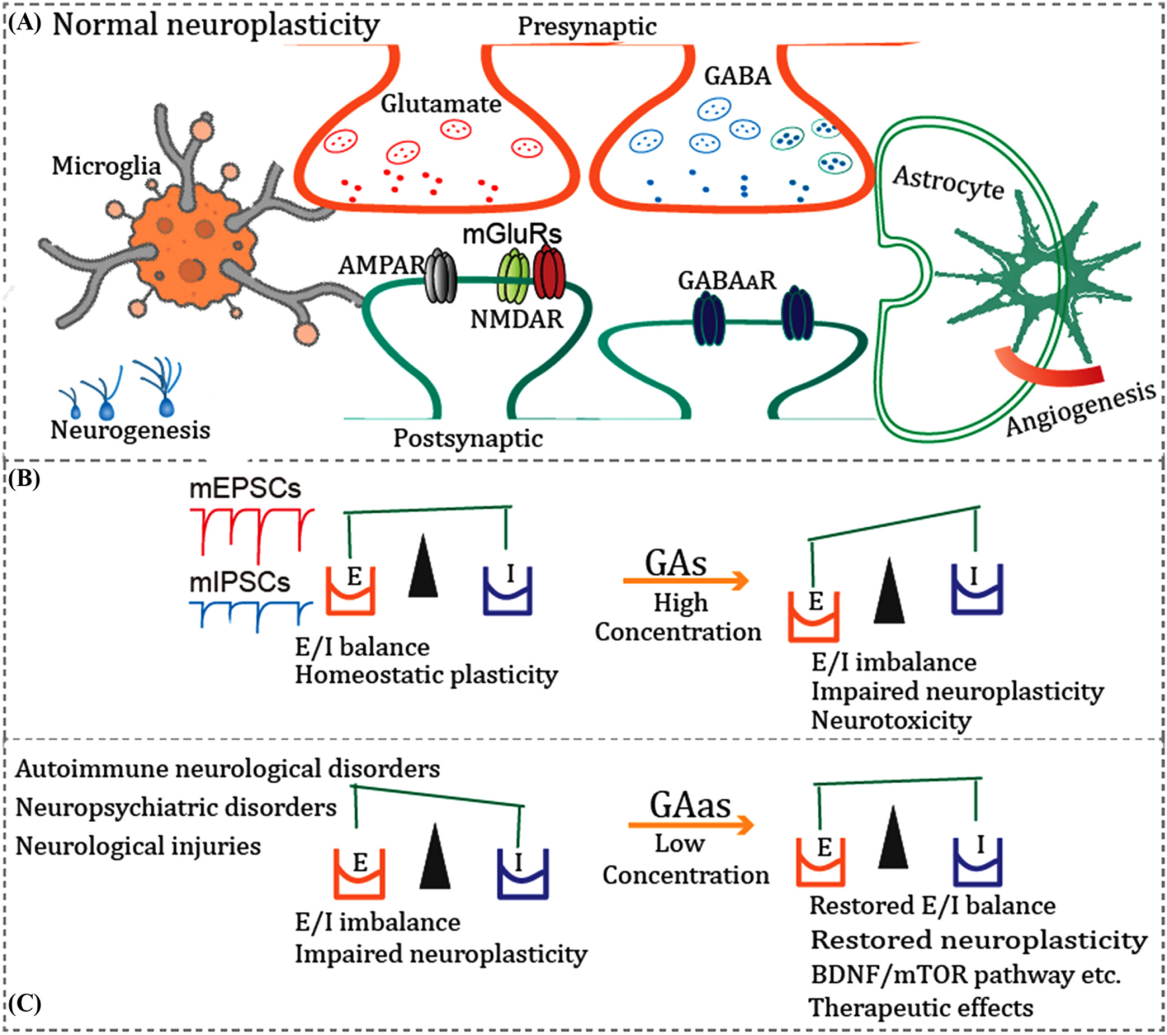
Conceptual mechanism of neurotoxicity and therapeutic potential of general anesthetics (GAs) used in this review. Graphic representation of neuroplastic mechanisms under developmental neurotoxicity and potential therapeutic effects of GAs. (**A**) Neuroplasticity comprises changes in plasticity-related receptor and molecular pathways, neurogenesis, angiogenesis, gliogenesis, dendritogenesis, neurotransmitter release, and formation of connections. (**B**) Neuroplastic mechanisms of developmental neurotoxicity caused by high concentration/dose GAs. (**C**) Neuroplastic mechanisms under potential therapeutic effects of GAs for mental illness characterised by abnormal neuroplasticity as core pathologies, such as autoimmune neurological disorders, neuropsychiatric disorders, and neurological injuries. **Abbreviations**: AMPAR, α-amino-3- hydroxy-5-methyl-4-isoxazolepropionic acid receptor; E/I, Excitatory/inhibitory; GABA, γ-aminobutyric acid; GABA_A_R, Type A GABA receptor; NMDAR, N-methyl-D-aspartate receptor; mGluRs, metabotropic glutamate receptors; mEPSC, miniature excitatory synaptic current; mIPSC, miniature inhibitory postsynaptic current; GAs, General Anesthetics.

Most of the clinical trials mentioned above are mainly based on the revealed pathology of neuropsychiatric disorders and the fact that GAs are potent modulators of neuroplasticity. Indeed, the identified pathways underlying the GAs for the therapy of neuropsychiatric disorders, as revealed by various animal models, intersect with mechanisms regulating synaptogenesis and synaptic structural plasticity, as well as excitatory-inhibitory imbalances in neural circuits [139, 144, 145]. Of these, the antidepressant effects of ketamine are the most extensively studied including activation of the AMPAR, opioid receptor system, mTORC1 signaling, glycogen synthase kinase-3, and BDNF-TrkB signaling, enhancing GABAergic synaptic inhibition, inducing vascular endothelial growth factor, protein p11 and synaptic proteins, inhibition of hyperpolarization-activated cyclic nucleotide-gated channel, regulation of neurotransmitter release, modulation of microRNAs and gut microbiota [162, 163, 201-206]. The mechanisms of other GAs (*e.g*., sevoflurane and propofol) for the treatment of neuropsychiatric disorders are similar to the antipsychotic mechanisms of ketamine [207-210]. For example, several studies have shown that GAs modulate the release and metabolism of dopamine and 5-hydroxytryptamine, which may be one of their important mechanisms of antipsychotic action [211-214]. Specifically, Zhu XN *et al*. found that propofol binds directly and potently to the dopamine transporter (DAT), inducing rapid and sustained dopamine accumulation in the nucleus accumbens (NAc). The increased dopaminergic tone then drives a biased activation of dopamine receptor-1-expressing medium spiny neurons in the NAc, which contributes to the rapid relief of anhedonia in chronically stressed animals [215]. In summary, preclinical and clinical evidence support the restoration of neuroplasticity as a convergent downstream mechanism of antipsychotic action of GAs (Fig. **1**). GAs modulate synaptic plasticity and neurotransmission through the various pathways described above to restore adaptive rewiring of pathological neurocircuitry, thereby providing a neuroplasticity-focused framework to explain the therapeutic effects of GAs.

### 4.2 Neuroprotection and Therapeutic Potential in Autoimmune Neurological Disorders

Autoimmune neurological disorders often have multifocal symptoms and typically present subacutely. Multiple structures in the central and peripheral nervous systems can be targets of pathogenic autoantibodies, leading to diverse clinical presentations. Manifestations in CNS include neuropsychiatric symptoms, seizures, encephalopathy, catatonia, sleep disorders, chorea, myoclonus, optic neuritis, opsoclonus, stiff person syndrome, brainstem syndromes, cerebellar ataxia, and myelopathy [216-218]. Peripheral nervous system (PNS) symptoms include radiculopathy, plexopathy, peripheral neuropathy, neuronopathy, neuromuscular junction disorders, and muscle disorders. Autonomic nervous system manifestations include anhidrosis, difficulty transitioning from dark to light, cardiac arrhythmias, gastrointestinal dysmotility, orthostasis, and urologic dysfunction [218, 219]. The “classical” neurological autoimmune diseases affecting either the CNS or the PNS, such as stiff-person syndrome, multiple sclerosis (MS) and Amyotrophic lateral sclerosis (ALS) [219]. At present, traditional immunotherapies are the mainstay of treatment, but randomized controlled studies are lacking. The current lack of reliable and effective treatments puts these patients in a difficult situation which call for new therapeutic directions. Based on diseases characterized by neuronal and synaptic loss, resulting in an imbalance of excitatory and inhibitory synaptic transmission and consequently impairment of neural function and clinical presentations, neuromodulators such as GAs, might be a potential for the development of new therapies for autoimmune diseases [220]. For example, increasing GABAergic activity ameliorates ongoing paralysis in encephalomyelitis by decreasing MAPK signaling and diminishing subsequent adaptive inflammatory responses to myelin proteins in an animal model of MS [221].

2 h of 2.5% sevoflurane reduces clinical disease in a mouse model of MS [222], and 100 mg/kg propofol hemisuccinate given three times a day from day 7 or day 12 until day 16 after autoimmune encephalomyelitis (EAE) initiation, significantly reduced maximal EAE score in rat experimental EAE [223]. Moreover, currently, the only established drug for ALS is riluzole, a glutamate antagonist, which causes minor side effects in some patients with ALS and, very rarely, serious adverse events [224]. In clinical practice, several case reports suggest that general anesthetics have at least a palliative effect. For example, administration of IV propofol at modest doses produced immediate relief of spasms without sedation in patients with stiff-limb syndrome [225, 226]. This treatment provided an effective therapeutic bridge and innovative therapeutic direction. Although there is little research in this area at the moment, that doesn't stop it from being a promising direction for drug development.

## CONCLUSION AND PERSPECTIVES

The advent of GAs has greatly advanced human medicine, allowing increasingly complex surgical and diagnostic procedures to be performed safely. However, GAs-induced developmental neurotoxicity is generally accepted, but this has not been replicated convincingly in clinical studies. GAs powerfully modulate developing neuroplasticity and have been shown to cause long-lasting ultrastructural and functional changes in the developing brain. While this plasticity poses a risk, it also creates a therapeutic window. The non-anesthetic effects of GAs, particularly in the field of neuropsychiatric disorders, have been recognized and used in clinical practice since very early times. However, concerns about the potential neurotoxicity of GAs have at times overshadowed their protective and therapeutic potential, slowing the progress of related research. With the rapid increase in the incidence of mental illness and the limitations of existing clinical antipsychotics, the neuroprotective and therapeutic role of GAs has received renewed attention. Impaired neuroplasticity resulting in E/I imbalance in neural circuits is central to the pathogenesis of psychiatric disorders, and the robust modulation of neuroplasticity and synaptic activity by GAs raises the possibility of a potential therapeutic role for these GAs in altering mental states. Based on this hypothesis, a growing number of clinical reports have demonstrated the psychotherapeutic effect of various GAs in patients with neuropsychiatric disorders and have extensively elucidated the molecular and cellular mechanisms underlying this effect over the last decades. This opens new possibilities for treating neurological injuries and neuropsychiatric disorders, as well as new research opportunities for anesthesia providers. However, the translation of these findings into routine clinical practice is mainly challenging. Many therapeutic applications of GAs require multidisciplinary collaboration and the involvement of anesthesiologists to manage respiratory and cardiovascular functions, which increases the complexity and cost of treatment.

Furthermore, key questions remain unresolved, including optimal dosing, administration schedules, and treatment durations to maximize therapeutic benefits while minimizing risks. To advance the field, future research should focus on large-scale, rigorously designed clinical trials to validate the efficacy of GAs in specific neuropsychiatric and neurological contexts. Additionally, efforts should be directed towards refining delivery methods to enhance accessibility and cost-effectiveness. As we continue to unravel the mechanisms by which GAs influence neuroplasticity and mental states, this area of research has the potential to transform the landscape of psychiatric and neurological treatment, offering anesthesia providers exciting opportunities to expand their roles in multidisciplinary care.

## Figures and Tables

**Table 1:** Clinical trials of GAs (beyond ketamine) for therapy of neuropsychiatric disorders.

**Author/** **Year**	**Patients**	**No. of Patients**	**Study Design**	**Groups**	**GAs**	**Dose and Duration**	**Outcome**
Miller DH/1953	Catatonic schizophrenia	30	3-week, RCT	ECT vs. Pentothal vs. Pentothal plus NET	Pentothal	7.5 g IV for 5 times weekly for 4 weeks	Improvement in social performance does not vary significantly with the various types of treatment [150]
Lambourn J/1978	Depressive psychosis	16	2-week, RCT	ECT plus GAs VS. simulated ECT plus GAs	Methohexitone	70mg IV, 3 times weekly for two weeks	No difference in the HAMD rating scale was found between the two groups under double-blind conditions [151]
Johnstone *et al.* 1980	Endogenous depression	70	RCT	ECT plus GAs vs. Simulated ECT plus GAs	Methohexitone	1.5 mg/kg IV, eight treatments over 4 weeks	The difference was small relative to the considerable improvement of both groups over the 4-week treatment period [152]
Freeman *et al.* 1978	Patients with depressive illness	40	Double-blind controlled trial	ECT plus GAs vs. Simulated ECT plus GAs	Thiopentone	150-300 mg IV, twice weekly	ECT was significantly superior to simulated ECT in the treatment of depressive illness [153]
Langer *et al.* 1985	Treatment-refractory depressed patients	11	Open explorative study	Single group	Isoflurane and nitrous oxide	Anesthesia was initiated with thiopental and maintained with nitrous oxide, and isoflurane (2-4%) to maintain ES for 3-5 min for several treatments (1 to 6 times)	Rapid antidepressant effects were observed in 9 out of 11 patients (*p* < 0.0001). Effects were reproducible and lasted up to several weeks [154]
Carl *et al.* 1988	Treatment-refractory depressed patients	10	Open comparative study	Anesthetic Therapy vs. ECT	Isoflurane	Anesthesia was induced with Thiopentone and maintained with isoflurane up to 4% in N2O/O2 to maintain Burst-suppression for 4-10 min for six treatments	Anesthetic therapy with Isoflurane could produce results similar to ECT in Treatment-Refractory Depressed Patients [155]
Langer *et al.* 1995	Drug-refractory, severely depressed women	20	Double-blind, controlled comparison	Isoflurane vs. ECT	Isoflurane	Isoflurane started at 4% until the first EEG suppression, then reduced to 2.5% until 15 min of predominant burst suppression, six sessions, twice weekly.	The isoflurane group showed significant rapid antidepressant effects and cognitive improvement. ECT also showed antidepressant effects, but with cognitive deterioration. ISONAR patients improved further during follow-up, while ECT patients tended to relapse [156]
Weeks *et al.* 2013	Treatment-refractory depressed patients	28	Open-label, two-arm treatment trial	ECT vs. Isoflurane	Isoflurane	ECT: 8-12 sessions over 2.5-3.5 weeks. 4% Isoflurane: 10 sessions over 3 weeks	Both treatments produced significant reductions in depression scores 4 weeks after treatment [157]
Nagele P *et al.* 2015	Treatment-Resistant Major Depression	20	A Proof-of-Concept Trial	Single group	Nitrous oxide	50% nitrous oxide for one hour	Nitrous oxide has a rapid and marked antidepressant effects [165]
Nagele P *et al.* 2021	Treatment-resistant depression	24	A phase 2 trial	(i) 50% nitrous oxide, (ii) 25% nitrous oxide, or (iii) placebo (air/oxygen)	Nitrous oxide	25%, 50% Nitrous oxide	25% nitrous oxide has comparable efficacy to 50% nitrous oxide in improving treatment-resistant depression [167]
Mickey BJ *et al.* 2018	Treatment-resistant depression	10	A Pilot Study	Single group	Propofol	Propofol was dosed to strongly suppress electroencephalographic activity for 15 minutes for a series of10 infusions	Propofol may trigger rapid, durable antidepressant effects similar to electroconvulsive therapy but with fewer side effects [170]
Zhao T *et al.* 2022	Schizophrenia	10	A Pilot Study	Single group	Sevoflurane	1% sevoflurane 5 hrs per day for 6 days	1% sevoflurane effectively alleviated schizophrenia patients' symptoms [172]

**Abbreviations**: Nonconvulsive electrotherapy, NET; Electrocerebral silence, ES; Hamilton Rating Scale for Depression, HAMD; Neuropsychiatric disorders, NPD; RCT, Randomised controlled trial.

## LIST OF ABBREVIATIONS

ADHD: Attention-Deficit/Hyperactivity Disorder
ALS: Amyotrophic Lateral Sclerosis
AMPA: Alpha-Amino-3-hydroxy-5-Methyl-4-isoxazolepropionic Acid
ASD: Autism Spectrum Disorder
BBB: Blood-Brain Barrier
BDNF: Brain-Derived Neurotrophic Factor
BDZs: Benzodiazepines
CaMK2: Ca^2+^ Dependent Calmodulin-dependent Protein Kinase
CGEs: Caudal Ganglionic Eminences
CMRO2: Cerebral Metabolic Rate of Oxygen
CNS: Central Nervous System
DAT: Dopamine Transporter
E/I: Excitation-Inhibition
EAE: Autoimmune Encephalomyelitis
ECT: Electroconvulsive Therapy
ERK: Extracellular Regulated Kinase
FDA: Food and Drug Administration
GABA_A_: GABA type A
GAS: General Anesthesia compared to Spinal Anesthesia
GAs: General Anesthetics
GD: Gestational Day
HIE: Hypoxic-Ischemic Encephalopathy
ID: Intellectual Disability
LTD: Long Term Depression
LTP: Long-Term Potentiation
MASK: Mayo Anesthesia Safety in Kids
MDD: Major Depressive Disorder
MGEs: Medial Ganglionic Eminences
MS: Multiple Sclerosis
NAc: Nucleus Accumbens
NDDs: Neurodevelopmental Disorders
NMDA: N-methyl-D-Aspartate
NPDs: Neuropsychiatric Disorders
PANDA: Pediatric Anesthesia NeuroDevelopment Assessment
PCD: Programmed Cell Death
PMA: Postmenstrual Age
PND: Postnatal Day
PNS: Peripheral Nervous System
TBI: Traumatic Brain Injury
